# Clinical Implications of Mechanical and Adhesive Differences Between Branded and Generic Tulobuterol Patches in Asthma Management

**DOI:** 10.7759/cureus.96638

**Published:** 2025-11-12

**Authors:** Atsushi Ishimura, HIna Tabayashi, Motoki Inoue

**Affiliations:** 1 Laboratory of Clinical Pharmacy Assessment, Hoshi University, Shinagawa, JPN; 2 Physical Chemistry, Hoshi University, Shinagawa, JPN

**Keywords:** adhesive property, asthma management, generic drug, mechanical testing, pharmaceutical formulation, transdermal drug delivery, tulobuterol patch

## Abstract

Aim of the study

This study aimed to evaluate the mechanical and adhesive properties of one branded and six generic tulobuterol transdermal patches commonly used for asthma treatment in Japan, and to clarify how differences in excipient composition influence patch usability and performance.

Materials and methods

A branded tulobuterol patch and six generic alternatives were analyzed. Mechanical strength was assessed using a uniaxial tensile tester, and adhesive performance (tackiness) was evaluated with a spherical probe detachment test. All measurements were conducted in quintuplicate under controlled temperature (25 ± 2°C) and humidity (60 ± 5% RH). Excipient compositions were identified from publicly available package inserts.

Results

Significant formulation-dependent differences were observed. The branded patch ruptured early under high stress with limited elongation, indicating rigidity, whereas several generics exhibited higher flexibility and elongation before rupture. Tackiness values also varied widely: some generics showed excessive adhesion, while others exhibited insufficient tackiness. These variations correlated strongly with differences in adhesive polymers and plasticizers.

Conclusion

Despite having equivalent pharmacokinetic profiles, branded and generic tulobuterol patches differ in their mechanical and adhesive characteristics, which may influence clinical usability and patient adherence. The results emphasize the importance of considering excipient-driven performance when selecting transdermal products for asthma management.

## Introduction

The global burden of healthcare costs continues to rise, driven by advances in medical technology and the rapid aging of populations. In response, many countries have implemented cost-containment strategies that promote the use of generic drugs as substitutes for brand-name medications [1]. This policy shift aims to preserve the sustainability of national healthcare systems while ensuring broad access to essential pharmacotherapies [2]. However, concerns persist regarding the interchangeability of generic formulations, particularly for complex dosage forms such as transdermal drug delivery systems (TDDS) [3].

In Japan, the government has strongly advocated for the use of generics in its effort to control increasing medical expenditures, which exceeded ¥47 trillion in fiscal year 2023-the highest on record [4]. In October 2024, a new reimbursement system known as the “Specified Medical Care Coverage” was introduced, requiring patients to pay the price difference out of pocket when opting for brand-name drugs over reimbursed generics [5]. While this policy has significantly increased the uptake of generics, persistent issues have been reported. These include concerns regarding quality assurance and supply chain stability [6], pharmacokinetic variability among generics [7], and limited transparency about formulation-specific information [8].

Generic drugs are typically approved based on equivalence in active pharmaceutical ingredients (APIs) and fundamental quality parameters, allowing them to bypass full-scale clinical trials after the originator patent expires [9]. However, many generics incorporate alternative formulation technologies, especially when formulation patents remain in effect. In TDDS, differences in adhesive polymers, plasticizers, and other excipients can markedly influence skin compatibility, mechanical behavior, and drug release kinetics [10,11]. Notably, excipients are not strictly regulated for physicochemical equivalence in transdermal systems, despite their critical role in usability and patient safety [12]. In some cases, excipients used during manufacturing may remain only in trace amounts in the final product, at levels too low to exert pharmacological activity. Such excipients are not always listed in the package insert, which should be acknowledged when comparing formulations based on publicly available information.

Tulobuterol patches are one such example. Widely used in patients with bronchial asthma in Japan, they offer a non-invasive and once-daily treatment alternative for individuals who may have difficulty with inhalation therapy due to age, physical condition, or poor coordination. However, variations in excipient composition among generics may affect patch adhesion and flexibility of the drug matrix, all of which may impact clinical usability, such as patch retention during sleep, ease of removal, and potential for skin irritation. Excessively adhesive patches may cause discomfort or skin damage during removal, whereas insufficient adhesion may result in premature detachment during physical activity, particularly in active children [13]. Furthermore, patient and caregiver impressions of external patches have been shown to affect treatment preference and adherence [14]. Given these factors, pharmacists and clinicians must consider not only bioequivalence but also formulation-dependent attributes when selecting or substituting tulobuterol patches. Although crystallinity in branded tulobuterol patches has been previously documented [15], the extent to which differences in excipient composition among generics contribute to variations in mechanical properties has not been quantitatively characterized. Therefore, the primary objective of this study was to compare the mechanical and adhesive performance of branded and generic tulobuterol patches. The secondary objective was to explore the clinical implications of formulation-dependent differences in excipient composition on usability and patient adherence.

## Materials and methods

Study design and formulations

This study evaluated one branded tulobuterol transdermal patch (2 mg) and six generic products (designated as formulations A-F) commercially available in Japan. All patches were obtained from the same production lot and stored under controlled conditions (25 ± 2°C, 60 ± 5% RH); therefore, the observed variance represents measurement reproducibility rather than lot-to-lot variation. Excipients were identified from publicly available package inserts and summarized in Table 1.

**Table 1:** List of excipients used in branded and generic tulobuterol tape formulations based on publicly available package inserts ✓: Excipients included in the formulation; –: Not included

Excipients	Branded	Generic					
		A	B	C	D	E	F
polyisobutylene	✓	-	-	-	-	-	-
polybutene	✓	-	-	-	✓	-	✓
alicyclic saturated hydrocarbon resin	✓	-	-	✓	✓	✓	-
2-ethylhexyl acrylate, 2-ethylhexyl methacrylate, dodecyl methacrylate copolymer	-	✓	✓	-	-	-	-
squalane	-	✓	-	-	-	-	-
isopropyl palmitate	-	✓	-	-	-	-	-
octyldodecanol	-	-	✓	-	-	-	-
styrene-isoprene-styrene block copolymer	-	-	-	✓	✓	✓	✓
hydrogenated rosin glycerin	-	-	-	✓	-	-	-
oleic acid	-	-	-	-	✓	✓	-
dibutylhydroxytoluene	-	-	-	-	✓	-	-
liquid paraffin	-	-	-	-	✓	✓	✓
polyisoprene	-	-	-	-	-	✓	-
isopropyl myristate	-	-	-	-	-	✓	✓

Lot numbers: Branded: 64705YQ1 (Exp. 2026.3), Generic A: B515T (Exp. 2026.4), Generic B: A0133 (Exp. 2027.6), Generic C: 4C020 (Exp. 2026.2), Generic D: X24315 (Exp. 2027.4), Generic E: 240901 (Exp. 2026.8), Generic F: 4B17T (Exp. 2026.1)

Tensile property testing

Mechanical properties were assessed using a uniaxial tensile tester (TA_XTplusC, Stable Micro Systems) with A/TG grips. In this study, mechanical properties refer to the tensile behavior of the patch, including stress at rupture and elongation, which reflect its flexibility and structural integrity during application and wear. Each sample (10 mm × 50 mm) was secured and stretched at a rate of 2 mm/s until rupture or a maximum extension of 50 mm or 12 kg load. Stress (N) and displacement (mm) were recorded. Testing was performed in quintuplicate at 25 ± 2°C and 60 ± 5% RH.

Tackiness measurement

Tackiness was measured using a film-support rig (heavy duty platform/film support rig (HDP/FSR)). A 3-mm spherical stainless-steel probe applied a 100 g load at 0.5 mm/s for 10 seconds and was retracted at 10 mm/s. The peak detachment stress (N) was recorded. Each measurement was repeated five times under the same environmental conditions.

Statistical analysis

Data are presented as mean ± standard deviation (SD). Inter-formulation differences were analyzed using one-way ANOVA followed by Tukey’s post hoc test, with p < 0.05 considered statistically significant.

Ethical considerations

As this study used only commercially available pharmaceutical products and did not involve human or animal subjects, no ethical approval was required.

## Results

Mechanical properties

**Figure 1:**
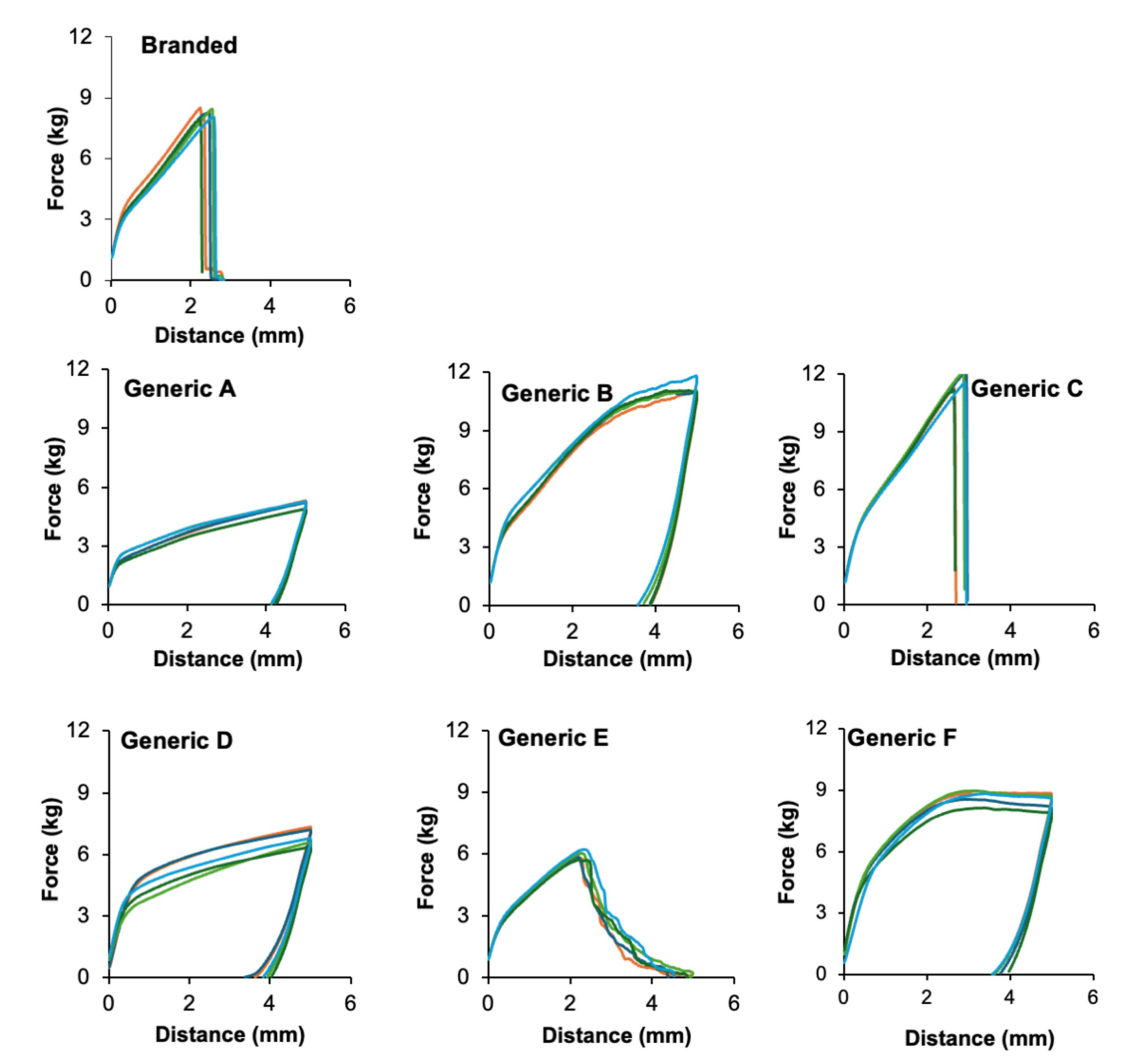
Tensile properties of tulobuterol tape formulations measured using a uniaxial tensile tester

All measurements were performed in quintuplicate, and results are expressed as mean ± standard deviation (SD). The objective of this study was to identify measurable differences among formulations rather than to determine statistical significance. Therefore, descriptive statistics were applied to visualize trends and reproducibility across samples. As summarized in Figure 1, tensile testing revealed clear formulation-dependent differences in mechanical behavior among the seven tulobuterol patches. The branded patch ruptured early under high stress with minimal elongation, indicating a rigid and brittle matrix. In contrast, most generic formulations exhibited a more gradual stress-strain profile and extended considerably before rupture, reflecting greater flexibility and elasticity of the polymer network.

Formulations A, B, D, and F did not rupture within the measurement range, suggesting high elongation tolerance and robust mechanical integrity. Formulation C exhibited a fracture pattern comparable to the branded patch but sustained greater elongation prior to failure, implying moderate flexibility. Formulation E demonstrated a distinctive failure mode in which the support film separated while the adhesive layer remained intact, consistent with poor interfacial adhesion or an excessively soft adhesive matrix. Overall, these results indicate that generic formulations generally favor flexibility over rigidity, which may improve skin conformity during application. However, excessive softness or interlayer weakness, as observed for formulation E, could compromise durability and ease of removal.

Tackiness

**Figure 2:**
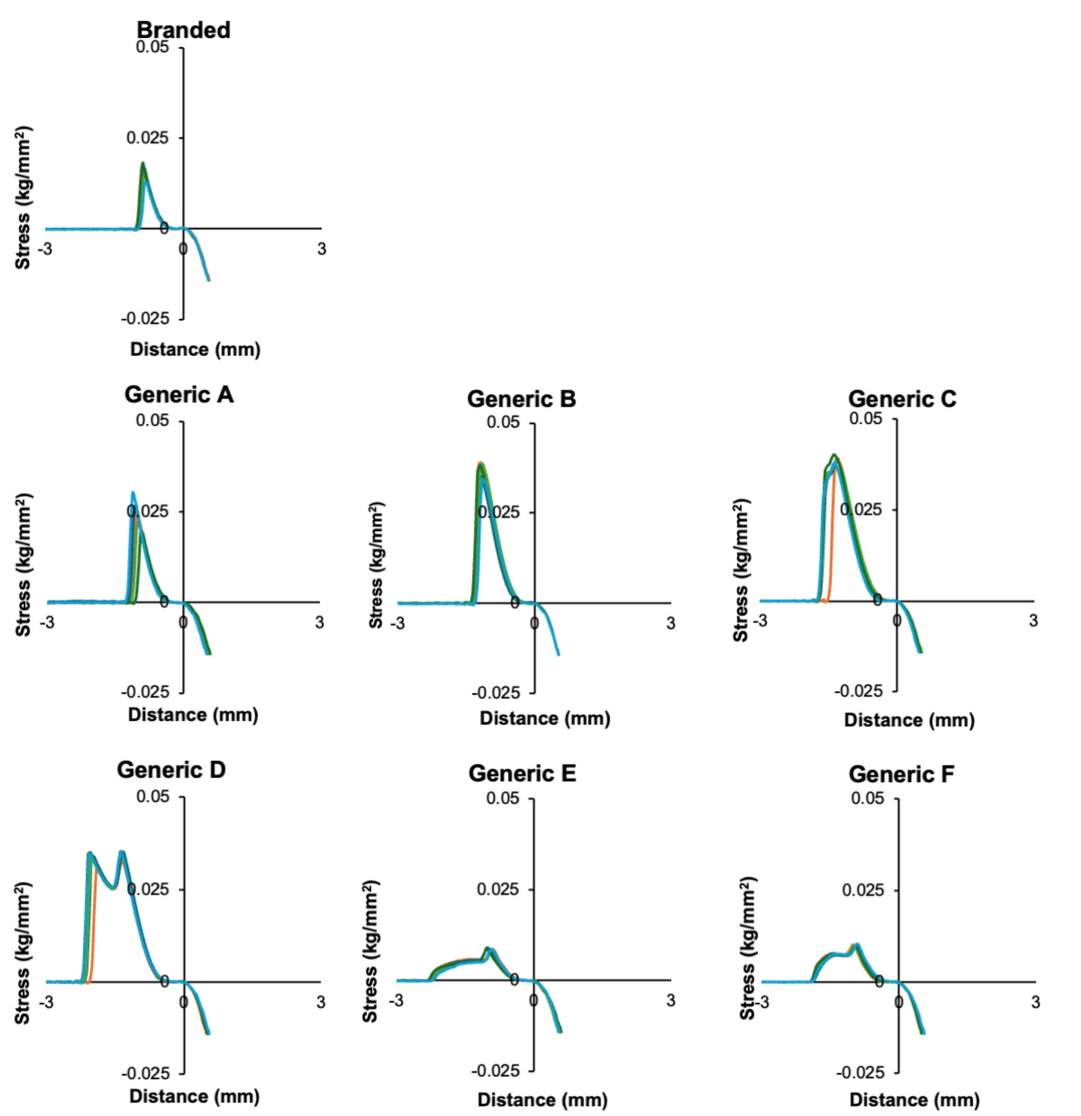
Adhesiveness of tulobuterol tape formulations evaluated using a film support rig

Quantitative evaluation of adhesive strength (Figure 2) also demonstrated pronounced variability among formulations. Generics A-D exhibited greater peak detachment stress than the branded patch, with formulations B and C exceeding it by more than 1.5-fold. Such strong adhesion could enhance patch retention under physical movement or perspiration, but may also increase the risk of discomfort or erythema during removal. Quantitative comparison of the maximum detachment stress (maximum stress) revealed clear formulation-dependent differences.

Formulations A-D exhibited significantly higher maximum stress than the branded patch (p < 0.01), while formulations E and F showed significantly lower values (p < 0.01, Welch’s t-test, n = 5). These differences correspond to the variations in adhesive polymers and plasticizers observed among formulations. The observed differences correlated closely with excipient composition, particularly the choice and ratio of adhesive polymers and plasticizers. Patches containing squalane and isopropyl palmitate tended to exhibit stronger adhesion, whereas those containing isopropyl myristate showed softer texture and reduced tackiness. Taken together, these findings highlight a formulation-dependent trade-off between adhesion and removability. Balancing these opposing characteristics is essential for optimizing both patient comfort and reliable drug delivery in transdermal therapy.

## Discussion

This study demonstrated clear formulation-dependent differences in the physicomechanical and adhesive behavior of branded and generic tulobuterol transdermal patches. Variations in the type and concentration of excipients were the most plausible drivers of these differences, as reflected by distinct stress-strain responses and detachment forces across products. The branded patch and Formulation C ruptured early with limited elongation, consistent with a rigid matrix, whereas Formulations A, B, D, and F exhibited greater elongation tolerance, indicative of enhanced flexibility. Formulation E presented a unique interfacial failure between the support film and adhesive layer, suggesting insufficient cohesion or an overly soft matrix. These observations align with prior reports showing that adhesive polymers, plasticizers, and vehicle composition govern both mechanical strength and skin adhesion in transdermal drug delivery systems (TDDS) [10-12]. Specifically, the stronger adhesion observed for formulations containing squalane and isopropyl palmitate, and the weaker adhesion associated with isopropyl myristate, correspond to known excipient effects on interfacial behavior and viscoelasticity [10-12]. From a clinical usability perspective, the trade-off between adhesion and removability observed here mirrors criteria proposed for evaluating generic TDDS, in which excessive tack can increase irritation risk while insufficient tack may lead to detachment [13]. These results complement previous physicochemical analyses of branded tulobuterol patches [15] by directly quantifying the relationship between excipient selection and mechanical flexibility and tackiness parameters, which are directly relevant to patient comfort and handling.

Taken together, the mechanical and adhesive profiles indicate a practical balance: formulations emphasizing flexibility may improve skin conformity but increase susceptibility to folding or deformation, whereas those emphasizing higher tack enhance retention but can cause discomfort upon removal. Although bioequivalence ensures comparable systemic exposure, it does not account for these usability attributes, which can influence adherence, particularly in children and older adults. Accordingly, pharmacists and clinicians should consider excipient-driven performance characteristics in parallel with pharmacokinetic equivalence when selecting or substituting tulobuterol patches.

This study has several limitations. First, all measurements were performed in vitro under controlled conditions without in vivo adhesion testing or clinical usability assessment, limiting external validity. Second, excipient information was obtained from publicly available package inserts and may exclude trace or proprietary components that could affect performance. Third, only one production lot per product was tested, so lot-to-lot variability was not evaluated. Future studies incorporating skin-permeation tests, sensory/handling evaluations, and patient-reported outcomes are warranted to confirm the clinical relevance of these formulation-dependent differences.

At present, no established clinical threshold exists for the minimum tackiness required to maintain 24-hour adhesion in tulobuterol or comparable TDDS formulations. Previous reports have proposed approximate ranges (0.2-0.4 N) for patches of similar size; however, these values vary depending on the formulation design and skin condition. Therefore, in this study, adhesive behavior was evaluated in relative rather than absolute terms, focusing on inter-formulation differences.

## Conclusions

Branded and generic tulobuterol transdermal patches exhibit marked differences in mechanical flexibility and adhesive behavior, primarily driven by variations in excipient composition. These formulation-specific differences influence patch usability, patient comfort, and the likelihood of maintaining reliable skin adhesion during daily activities. Generics designed with greater flexibility may improve skin conformity but risk deformation or premature detachment, whereas stronger adhesion can enhance retention yet increase discomfort upon removal. These findings underscore that the practical performance of transdermal drug delivery systems depends not only on pharmacokinetic equivalence but also on achieving an appropriate physicomechanical balance among their components. Optimizing excipient selection in generic TDDS formulations is therefore essential to ensure consistent therapeutic outcomes and patient adherence. Further clinical and usability studies are warranted to translate these in vitro insights into evidence-based product selection and patient guidance in asthma management.
